# Convergent evolution of cysteine-rich proteins in feathers and hair

**DOI:** 10.1186/s12862-015-0360-y

**Published:** 2015-05-07

**Authors:** Bettina Strasser, Veronika Mlitz, Marcela Hermann, Erwin Tschachler, Leopold Eckhart

**Affiliations:** Research Division of Biology and Pathobiology of the Skin, Department of Dermatology, Medical University of Vienna, Lazarettgasse 14, 1090 Vienna, Austria; Department of Medical Biochemistry, Medical University of Vienna, Vienna, Austria

**Keywords:** Feathers, Evolution, Epidermis, Epidermal differentiation complex, Keratin-associated protein, Cysteine-rich protein

## Abstract

**Background:**

Feathers and hair consist of cornified epidermal keratinocytes in which proteins are crosslinked via disulfide bonds between cysteine residues of structural proteins to establish mechanical resilience. Cysteine-rich keratin-associated proteins (KRTAPs) are important components of hair whereas the molecular components of feathers have remained incompletely known. Recently, we have identified a chicken gene, named epidermal differentiation cysteine-rich protein (EDCRP), that encodes a protein with a cysteine content of 36%. Here we have investigated the putative role of EDCRP in the molecular architecture and evolution of feathers.

**Results:**

Comparative genomics showed that the presence of an EDCRP gene and the high cysteine content of the encoded proteins are conserved among birds. Avian EDCRPs contain a species-specific number of sequence repeats with the consensus sequence CCDPCQ(K/Q)(S/P)V, thus resembling mammalian cysteine-rich KRTAPs which also contain sequence repeats of similar sequence. However, differences in gene loci and exon-intron structures suggest that EDCRP and KRTAPs have not evolved from a common gene ancestor but represent the products of convergent sequence evolution. mRNA *in situ* hybridization demonstrated that chicken EDCRP is expressed in the subperiderm layer of the embryonic epidermis and in the barbule cells of growing feathers. This expression pattern supports the hypothesis that feathers are evolutionarily derived from the subperiderm.

**Conclusions:**

The results of this study suggest that convergent sequence evolution of avian EDCRP and mammalian KRTAPs has contributed to independent evolution of feathers and hair, respectively.

**Electronic supplementary material:**

The online version of this article (doi:10.1186/s12862-015-0360-y) contains supplementary material, which is available to authorized users.

## Background

The evolution of genes that facilitate the cornification of keratinocytes was crucial for the evolution of skin appendages such as hair and feathers. Mature skin appendages consist of dead keratinocytes which are interconnected by stable junctions and filled with highly cross-linked proteins. The process of intracellular protein cross-linking involves either transglutamination, the covalent connection of glutamine and lysine residues, or disulfide bonding, that is, the covalent connection of cysteine residues. Mammals have distinct sets of proteins that have evolved as efficient substrates for cornification-associated cross-linking [1]. These cornification substrates include cysteine-rich keratins, also known as hair keratins [2,3], and keratin-associated proteins (KRTAPs) [4,5] as well as proteins encoded by genes of the so-called epidermal differentiation complex (EDC) [6,7]. The latter is a cluster of genes that are expressed during terminal differentiation of epidermal keratinocytes. Many proteins encoded by EDC genes contain glutamine and lysine-rich sequence motifs and some of them also have a high cysteine content around 15% [6]. Recently, we have reported that sauropsids (reptiles and birds) have genes homologous to hair keratin genes [3] as well as a gene cluster homologous to the mammalian EDC [8]. However, homologs of KRTAPs have not been identified in sauropsids [3].

The EDC of the chicken contains a gene coding for a protein with an extremely high content of cysteine residues, named epidermal differentiation cysteine-rich protein (EDCRP) [8]. Cysteine makes up 140 of the 385 amino acid residues of chicken EDCRP. The *EDCRP* gene and its neighboring genes have 2 exons, of which the second one contains the entire coding region [8]. Thus, EDCRP has the same gene structure as the genes encoding the so-called beta-keratins (also known as corneous beta-proteins) [8], which are the most abundant proteins of sauropsidian scales and claws as well as avian feathers [9,10]. Expression of *EDCRP* was detected by RT-PCR screening in embryonic skin from various body sites of the chicken [8]. However, its expression pattern at the cellular level has remained elusive.

Here we report the investigation of the evolutionary history and the expression pattern of EDCRP in the skin and feathers of the chicken. Our data suggest an important role of EDCRP in the molecular architecture and in the evolution of feathers.

## Results

### EDCRP is expressed in subperiderm and feathers of the chicken

**Figure 1:**
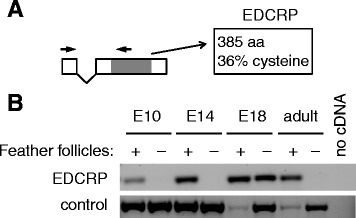
EDCRP is expressed in the skin containing feather follicles and in late embryonic skin of the chicken. **(A)** Schematic depiction of the chicken EDCRP gene. White boxes indicate exons, and grey shading marks the coding region. Arrows indicate the position primer annealing sites for the amplification of cDNAs. **(B)** Skin from the wings, containing feather follicles (+), and skin from the lower part of the legs, lacking feather follicles (−), was prepared from embryonic (days E10, E14 and E18) and adult chicken. RNA was extracted and reverse-transcribed to cDNA, which was subjected to PCRs specific for EDCRP and a control gene (caspase-3). PCR without template cDNA was performed as negative control reaction.

Based on our previous analysis of the gene structure of chicken *EDCRP* [8], we designed primers and probes suitable for the specific detection of EDCRP mRNA by RT-PCR and *in situ* hybridization, respectively. RT-PCR was performed on RNAs from skin and skin appendages of chicken embryos and adult chicken (Figure 1). In the skin of the legs, EDCRP was detected on embryonic day E18 but not, at significant amounts, on days E10 and E14 nor in adult leg skin. By contrast, feather follicles and feathers were positive for EDCRP from E10 to adults.

**Figure 2:**
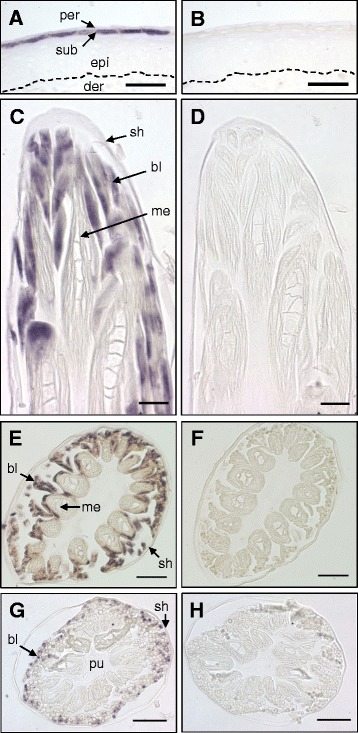
EDCRP is expressed in the subperiderm and the feathers of the chicken. Leg skin on embryonic day E18 **(A, B)** and growing feathers (E18) in longitudinal sections **(C, D)** and cross-sections **(E-H)** were subjected to *in situ* hybridization with either anti-sense probes specific for EDCRP mRNA **(A, C, E, G)** or sense probes as negative controls **(B, D, F, H)**. bl, barbules; der, dermis; epi, epidermis; me, medulla of a barb ridge; per, periderm; sh, feather sheath; sub, subperiderm; pu, pulp. Size bars, 40 μm **(A, B, E, F, G, H)**, 20 μm **(C, D)**.

To more precisely determine the expression pattern of *EDCRP*, we performed mRNA *in situ* hybridization. In the embryonic epidermis, EDCRP mRNA was absent from the basal and suprabasal epidermal layers that correspond to those of adult chicken skin and in the superficial embryonic skin layer, the periderm. By contrast, strong staining was present in the subperiderm (Figure 2A), a layer of embryonic periderm-related cells that is specific to archosaurs (Crocodilia and Aves) [11]. The negative control experiment in which the mRNA antisense probe was replaced by a labeled probe in sense orientation yielded no staining (Figure 2B), thereby confirming the specificity of the assay. In some regions of the embryonic skin, the subperiderm showed little or no labeling, which was likely caused by masking or degradation of EDCRP mRNA in advanced cornification of the subperiderm.

**Figure 3:**
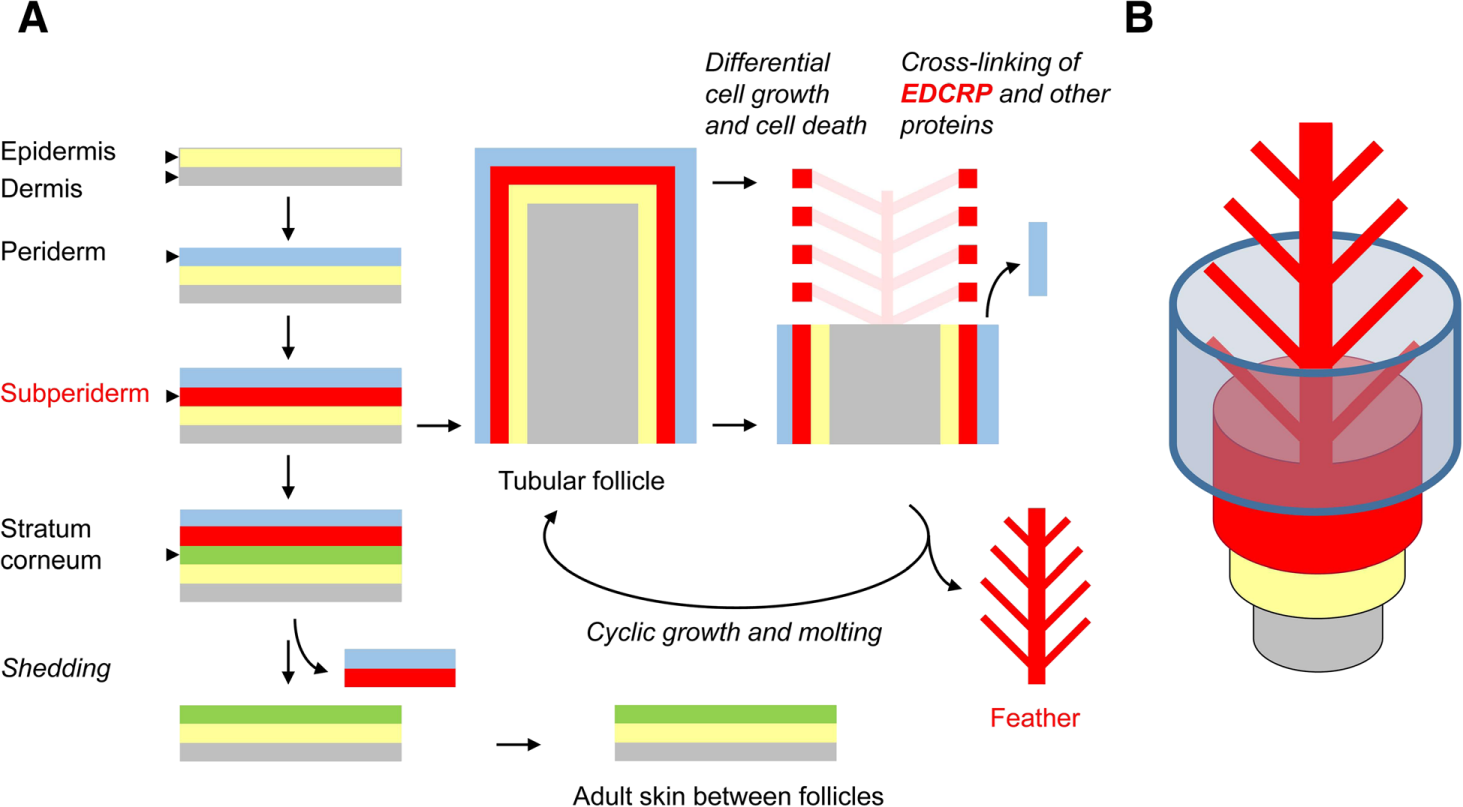
Embryonic epidermal stratification and expression of EDCRP are maintained in feather follicles. **(A)** The skin layers during embryonic development and within a feather follicle are shown schematically. The topology of the layers is maintained. The permanent structures of feathers are made by cells that correspond to the embryonic subperiderm whereas other cells of the feather follicle degenerate during the feather morphogenesis process. The development of feathers within the feather follicle is repeated in the adult animal. Note that the cross-section through the tubular feather follicle shows feather elements (red squares) that are connected by barbs (indicated by light red color) located outside of the plane of section. **B)** Three-dimensional depiction of the topology of epithelial layers in the feather follicle. The colors show equivalence of the feather follicle layers to the embryonic skin layers in panel **A**.

*In situ* hybridization also revealed prominent expression of *EDCRP* in barbule cells of the feathers. Positive labeling was observed in samples of E15 (not shown) and on E18 (Figure 2C, E, G). The labeling was strongest in the developmentally youngest barbule cells in the lateral part of the feather (Figure 2C, E, G) whereas cornified barbs were not stained. Negative control experiments with sense probes confirmed the specificity of the signals (Figure 2D, F, H). The feather sheath and feather pulp were consistently negative (Figure 2C-H). The expression pattern of EDCRP is consistent with the hypothesis that the cyclical growth and shedding of feathers is a modified replication of a series of steps in embryonic skin development (Figure 3). In this model, the feather sheath is the equivalent of the embryonic periderm, as suggested by the common expression of scaffoldin and presence of periderm granules [12] (blue layers in Figure 3); the permanent components of the feathers are equivalent to the embryonic subperiderm with both expressing EDCRP (red in Figure 3); and the epithelial cell layer, that borders on the dermis (grey layers in Figure 3) during early feather development and later degenerates [13], is equivalent to the epidermis *proper* of the embryo (yellow layers in Figure 3). EDCRP appears to function both in the subperiderm and in the feathers, presumably by facilitating intermolecular cross-linking via its many cysteine residues.

### The cysteine-rich sequence of EDCRP is conserved among birds

To test which features of chicken EDCRP are conserved among birds, we characterized EDCRP genes in a panel of genome sequences from phylogenetically diverse avian species and compared the sequences. Indeed, we identified partial or complete coding sequences of EDCRP in all birds investigated (Additional file 1: Table S1, Additional file 2: Figure S1). Gaps in the genome sequence assemblies and presumable artefacts of genome sequencing or sequence assembly caused gaps or premature ends in the coding sequence of EDCRP orthologs of several species (Additional file 2: Figure S1, and data not shown). A frameshift within the coding sequence of EDCRP was present in the genome sequence of the zebra finch deposited in the GenBank (Accession number NC_011489.1). However, amplification and sequencing of the zebra finch EDCRP gene revealed a contiguous open reading frame encoding all the protein domains present in chicken EDCRP (Additional file 2: Figure S1). The genome sequence of ostrich (*Struthio camelus australis*) contained a gap within the *EDCRP* gene. Amplification and sequencing of this region suggested the presence of 2 *EDCRP* forms, perhaps corresponding to 2 alleles, which differed by the absence or presence of a 27 bp stretch of nucleotides within a repetitive sequence region (Additional file 2: Figure S1). Thus, our data indicate that EDCRP is conserved among birds.

All available complete avian EDCRP genes contained a single coding exon that was preceded by a sequence highly similar to the experimentally verified non-coding exon 1 of chicken *EDCRP* [8]. Sequence comparison of exon 1 and the proximal promoter of phylogenetically diverse species of birds, including ostrich and tinamou from the basal clade Palaeognathae, showed high degrees of nucleotide sequence conservation (Additional file 3: Figure S2). A canonical TATA box, that is conserved in other avian and non-avian EDC genes [8] (Additional file 4: Figure S3), is replaced by the TATA-like element AATAAAA [14,15] in all avian *EDCRP* genes except for that of the loon (Additional file 3: Figure S2). This suggests that the evolution of the promoter of avian *EDCRP* might have involved a specific mutation replacing the ancestral TATA box with the TATA-like element and the reversion of this mutation in the loon.

**Figure 4:**
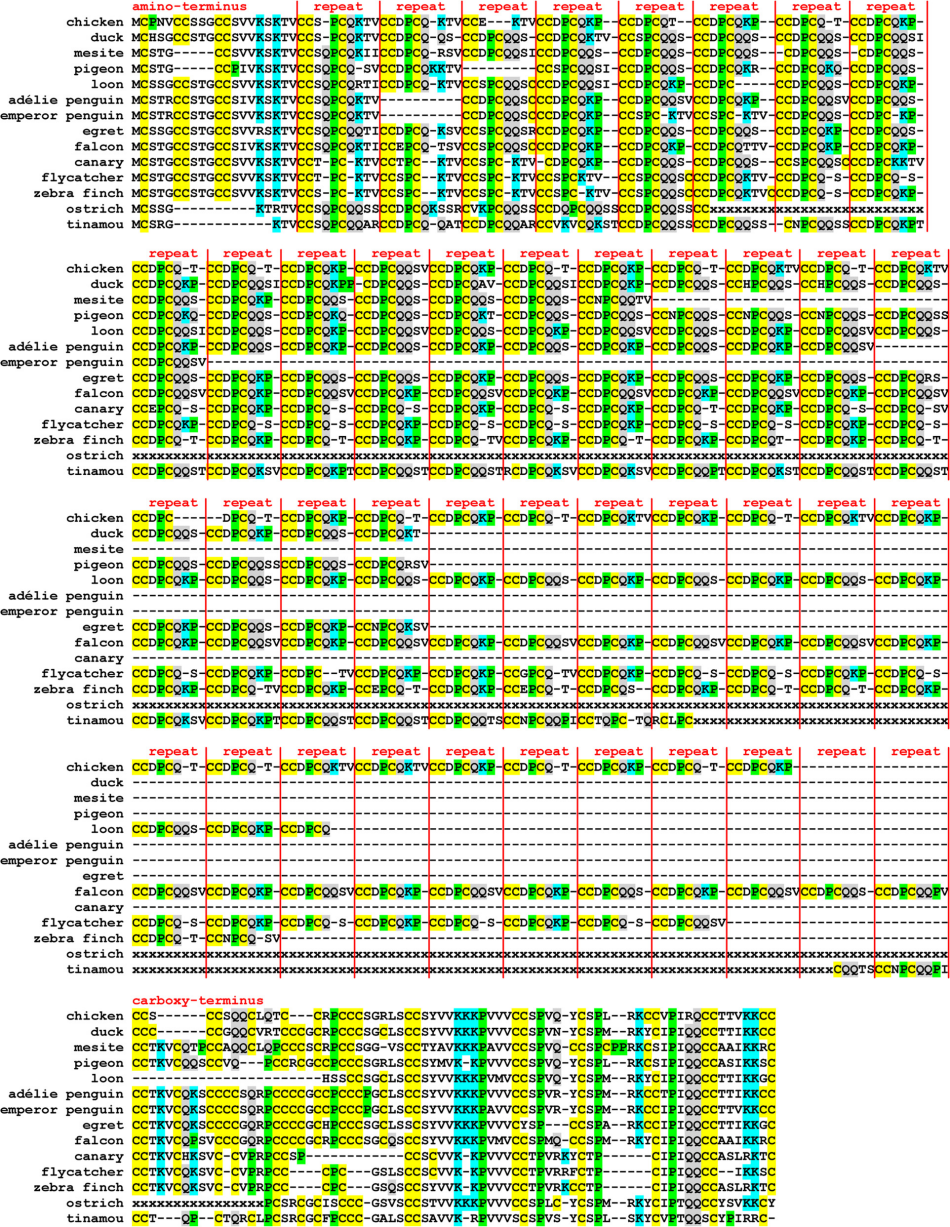
Avian EDCRPs contain conserved sequences at the amino-terminus and the carboxy-terminus as well as a variable number of conserved sequence repeats in the central segment. Amino acid sequences of epidermal differentiation cysteine-rich protein (EDCRP) from various bird species were aligned. Vertical lines separate the amino-terminus, the repeats of the central region and the carboxy-terminus. Hyphens were introduced to optimize the alignment. Color shading highlights the amino acid residues C, K, P and Q, which are assumed to be important for the function of the protein (see main text). x, amino acid residue missing because of gaps in genome sequences. Species: Adélie penguin (*Pygoscelis adeliae*), canary (*Serinus canaria*), chicken (*Gallus gallus*), duck (*Anas platyrhynchos*), egret (*Egretta garzetta*), emperor penguin (*Aptenodytes forsteri*), falcon (*Falco cherrug*), flycatcher (*Ficedula albicollis*), loon (*Gavia stellata*), mesite (*Mesitornis unicolor*), pigeon *(Columba livia)*, ostrich (*Struthio camelus australis)*, tinamou (*Tinamus guttatus*), zebra finch (*Taeniopygia guttata*).

The proteins encoded by *EDCRP* genes of different species vary in length (Additional file 1: Table S1) but have essentially the same basic organization in which a central segment containing multiple sequence repeats is flanked by amino-terminal and carboxy-terminal segments with unique sequences (Figure 4 and Additional file 2: Figure S1). The amino-terminal segment differs significantly between species of the basal avian clade Palaeognathae (ratites, e.g. ostrich, and tinamous) and Neognathae (all other birds), indicating an early evolutionary divergence in the structure of EDCRP. The carboxy-terminal segment shows a widely conserved basic organization which, however, appears to tolerate insertions and deletions of residues at several positions (Figure 4). The central region of EDCRP contains 6–56 repeats of 7–9 (and in exceptional cases 10) residues with the core sequence CCDPCQ. Each species has 2–4 types of repeat units that are defined by the residues on the carboxy-terminal side of the repeat core. The main repeat types are CCDPCQKP, CCDPCQK(T/S)V, CCDPCQ(T/S), and CCDPCQQS(V). In many, but not all, species the different repeat types are arranged in regular patterns. For example, the repeat units CCDPCQKP and CCDPCQQSV alternate 14 times in EDCRP of the saker falcon (*Falco cherrug*) (Additional file 2: Figure S1). The number of repeats shows high variability even among closely related species such as the penguins (Additional file 5: Figure S4). The amino-terminal and carboxy-terminal segments of EDCRP comprise 8–20 and 52–75 residues, respectively, and contain sequence motifs that are conserved among all birds investigated (Figure 4).

Despite the local sequence variabilities described above, all avian EDCRPs are characterized by a high content of cysteine (29-31% in Palaeognathae and 32-38% in Neognathae), suggesting that the capability of EDCRP to form disulfide bonds is important across birds. Of note, two consecutive cysteine residues (CC) are found at a periodicity of 8–11 residues (with exceptions) along the entire length of EDCRP proteins. In addition, glutamine and lysine residues, i.e. the target sites of transglutamination, are also abundant at conserved positions within EDCRP (Figure 4). The conserved presence of amino acid residues capable of protein crosslinking makes EDCRP highly competent to participate in the formation of mechanically resilient and hard epidermis-derived structures such as feathers.

### Phylogenetic analysis suggests independent evolution of EDCRP-like features in mammalian KRTAPs

**Figure 5:**
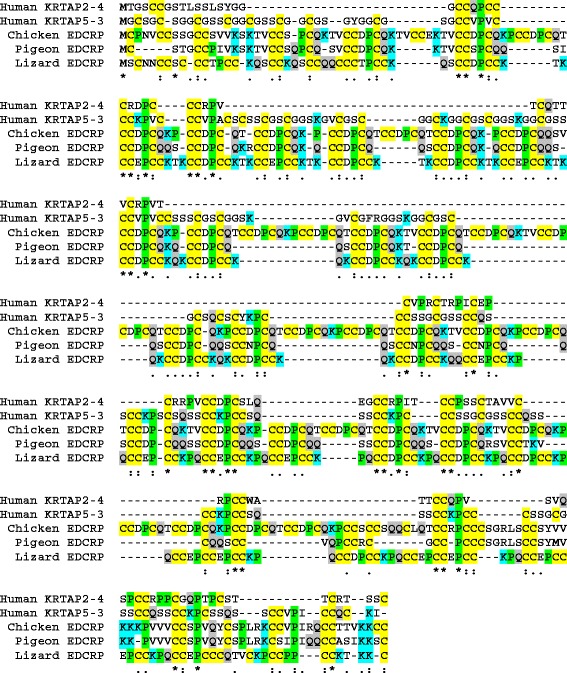
Amino acid sequence alignment of avian EDCRPs, lizard EDCRP and representative human cysteine-rich KRTAPs. Sequence similarity is indicated by the following symbols: “*”, conserved in 5/5 proteins; “:”, conserved in 4/5 proteins; “.”, conserved in 3/5 proteins. Color shading highlights the amino acid residues C, K, P and Q, which are assumed to be important for the function of the protein (see main text). Species: chicken (*Gallus gallus*), human (*Homo sapiens*), lizard (*Anolis carolinensis*), pigeon (*Columba livia*). KRTAP, keratin-associated protein.

The sequences of chicken, pigeon, and ostrich EDCRP were used as queries in tBLASTn searches for EDCRP-like genes in the genomes of non-avian vertebrates. Genes encoding proteins with both high cysteine content and sequences similar to that avian EDCRP were identified in the green anole lizard (*A. carolinensis*) and in mammals (Figure 5) but not in crocodilians (the sister group of birds), snakes (the sister group of the anole lizard), turtles, and anamniotes (fish and frogs). The EDCRP-like protein of the lizard was previously also termed EDCRP [8]. The mammalian proteins with EDCRP-like sequence motifs belong to the protein family of the KRTAPs [16]. The sequences of lizard EDCRPs and mammalian cysteine-rich KRTAPs are mostly similar to the central repetitive region of avian EDCRPs (Figure 5). However, some terminal sequence elements of avian EDCRP were also found in lizard EDCRP and KRTAPs (Figure 5). It is important to note that the sequences of all these proteins are dominated by a few amino acid residues, i.e. cysteine, proline, lysine, glutamine, and serine, so that complex sequence motifs are rare.

To further evaluate the likelihood that avian EDCRPs, lizard EDCRP and mammalian KRTAPs have a common ancestor, we compared their gene structures and flanking genes (synteny). Lizard *EDCRP* has the same gene structure (1 non-coding, 1 coding exon) as avian *EDCRP*s [8]. In contrast to the promoters of avian *EDCRP*s, the promoter of lizard *EDCRP* contains a canonical TATA box (Additional file 4: Figure S3). The lizard *EDCRP* gene is located at a similar position within the EDC as chicken *EDCRP*, i.e. between the conserved genes *EDWM* and the loricrin genes. Moreover, the orientation of the *EDCRP* genes relative to these genes is identical in birds and lizards [8]. These similarities are compatible with the hypothesis that *EDCRP*s of birds and lizards are orthologous. However, when we screened the genomes of other sauropsids (crocodilians, turtles, and snakes) for genes encoding *EDCRP*-like proteins, we did not find orthologs (our unpublished data). This suggests that this ancestral *EDCRP* gene, if it existed in a common ancestor of modern sauropsids, was lost in some of its descendants. Alternatively, genes of similar sequence may have emerged by convergent evolution in birds and lizards.

Mammalian *KRTAP* genes differ from avian *EDCRP* with regard to the exon-intron structure because *KRTAP*s have only a single exon. This exon is preceded by a promoter in which a TATA box is present. Unlike EDCRP genes, KRTAPs do not have a non-coding exon 1 [17]. The chromosomal locations of mammalian *KRTAP* genes are not syntenic with the sauropsidian *EDCRP* gene locus. In humans, more than 100 human *KRTAP* genes are distributed in 6 clusters on 4 different chromosomes (chromosome 2, 11, 17, and 21) with no *KRTAP* cluster being present in the EDC (chromosome 1). Actually, the human EDC does not have any gene in the region that corresponds to the locus of *EDCRP* genes in birds and the anole lizard, i.e. between *PGLYRP3* and *LOR* [8]. Similar *KRTAP* gene distributions are present in other mammals [18]. It is also important to note that *KRTAP*s diversified to encode proteins with high cysteine contents (similar to EDCRP) and proteins with low cysteine (but high glycine and tyrosine) contents [17].

**Figure 6:**
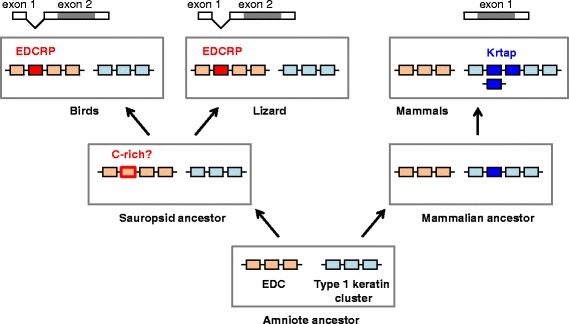
Differences in exon-intron structures and gene loci argue against common ancestry of EDCRP and KRTAPs. The exon-intron structures of EDCRP and KRTAP genes as well as their locations in the EDC and in the type 1 keratin gene cluster, respectively, are depicted schematically. Grey shading marks the coding region within exons. The inferred gene composition of ancestral genomes is shown below the schematics of the modern genomes. It remains uncertain whether the ancestral gene of avian and lizard EDCRPs was already cysteine (C)-rich.

**Figure 7:**
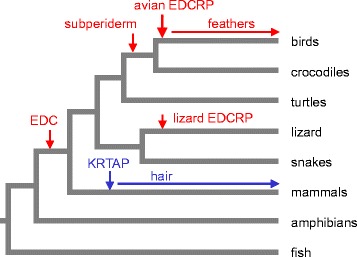
Scenario for the evolution of cysteine-rich proteins, feathers and hair. The putative origins of the epidermal differentiation complex (EDC), the subperiderm, avian and lizard EDCRPs as well as KRTAPs are indicated by vertical arrows on a schematic phylogenetic tree of vertebrates.

Together, the analyses of exon-intron structures and gene locus syntenies suggest homology of avian and lizard EDCRP but non-homology of these proteins to mammalian KRTAPs. The parsimonious evolutionary pathways leading to avian and lizard EDCRP as well as of mammalian KRTAPs are schematically depicted in Figure 6. Accordingly, sequence similarities between avian EDCRP and mammalian KRTAPs are likely to be the products of convergent evolution. Thus, the evolution of feathers and hair was associated with and perhaps facilitated by the independent origins of cysteine-rich structural proteins (Figure 7).

## Discussion

This study shows that birds have a protein with sequence similarity to cysteine-rich proteins of mammalian hair. The avian cysteine-rich protein is expressed in an archosaur-specific embryonic skin layer, the subperiderm, and in the feathers. Together, these findings shed new light into the cornification of cells that become the building blocks of feathers and allow to refine the hypotheses about the evolution and development of feathers [19-22].

Our genome screening has identified EDCRP homologs in all birds investigated and revealed conservation of sequence elements as well as considerable tolerance for insertions and deletions of amino acid residues at many positions. The central region of EDCRP consists of sequence repeats in which the amino-terminal part of the repeat unit is highly conserved whereas other residues are variable. In some species 2 types of repeats are arranged in an alternating pattern (Figure 4) whereas other species do not have this regular arrangement of different types of repeat units. The number of repeat units varied even among closely related species, such as the emperor penguin and the Adélie penguin (Additional file 5: Figure S4) [23]. Together, these data indicate that neither repeat regularity nor the length of the central region are critical for the function of EDCRP.

The most striking and most conserved feature of the EDCRP amino acid sequence is the highly biased abundance of individual amino acids. The relative cysteine content is among the highest of all proteins reported so far [24]. Only some mammalian KRTAPs have higher percentages of cysteine residues [18]. Moreover, EDCRP is rich in lysine and glutamine residues. While cysteine residues allow protein cross-linking via disulfide bonds, lysine and glutamine residues do so by undergoing transglutamination. Thus, this amino acid sequence qualifies EDCRP as an ideal cross-linking substrate during the cornification of keratinocytes. Cysteine residues are typically cross-linked during the formation of hard skin appendages such as claws/nails, hair and feathers, but to a much lower extent in the cornification of interfollicular epidermis or epidermal regions devoid of skin appendages, such as the soles [25]. Interestingly, a large portion of cysteine residues of EDCRP is present in the form of consecutive cysteine residues (CC) and notably, CCs are arranged in a regular pattern not only in the central repetitive region but also in the terminal segments. A similar pattern is present in many mammalian KRTAPs and has been proposed to facilitate protein cross-linking [16].

The results of this study suggest that the evolutionary origin of EDCRP occurred during the diversification of so-called simple EDC genes (SEDCs), which are genes comprising a 5′-terminal non-coding exon, one intron and a second exon in which the entire coding region resides [8]. An ancestral gene with this structure was likely already present in the last common ancestor of birds, reptiles and mammals. According to our hypothesis, duplications and sequence modifications of this primordial SEDC gene have led to the evolution of more than 20 SEDC genes in each chicken, lizard and humans [8]. Our data indicate that the evolution of EDCRP involved the replacement of an ancestral canonical TATA box by a TATA-like element, the loss of amino acid sequence motifs encoded by ancestral EDC genes [8], the accumulation of mutations that increased the cysteine content and the increase in the number of sequence repeat units in the central region by a mechanism such as inequal cross-over. From the presence of EDCRP in the avian species investigated it can be inferred that the time of origin of EDCRP has preceded the diversification of modern birds. Due to the common ancestry of all SEDC genes, avian EDCRP and lizard EDCRP have also evolved from a common ancestral gene which was present in the amniote cenancestor (see above) [8]. However, the question remains whether the sequence similarity between avian EDCRP and lizard EDCRP and their high cysteine content have been derived from a common ancestor or whether they appeared by convergent evolution. The assumption that a gene coding for a cysteine-rich EDCRP ancestor has been present in the ancestor of all modern sauropsids would imply that this gene (or its major sequence features) has been conserved only in the lineages leading to birds and lizards whereas it has been lost independently in the evolutionary lineages leading to 3 different clades of reptiles, namely crocodilians, turtles, and snakes, because none of the latter has an EDCRP homolog of comparable cysteine content. A more parsimonious explanation for the observed distribution of EDCRP among modern sauropsids is convergent evolution of the similar repeat units of avian and lizard EDCRPs from a common ancestor with lower cysteine content (Figure 6).

Convergent evolution is also the most likely mechanism for generating the sequence similarity between avian EDCRP and mammalian KRTAPs because the genes encoding EDCRP and KRTAPs are very likely to have different evolutionary origins. This notion is suggested by the difference in exon-intron structures (*EDCRP* has 2 exons whereas *KRTAP*s have 1 exon) and by the lack of gene locus synteny (*EDCRP* is located within the EDC whereas none of the *KRTAP* genes is located there) (Figure 6). A possible scenario, similar to a previously published hypothesis [26], for the evolutionary origin of *KRTAP* genes is the mutation of a keratin gene. *KRTAP* genes have the same organization as exon 1 of keratins, and both *KRTAP*s and the exon 1 of so-called “hair keratins” encode cysteine-rich amino acid sequences. Notably, there is strong evidence that the exon-intron structure of keratins and the increased cysteine content of “hair keratins” (which originally might have been claw keratins) have evolved prior to the divergence of the lineages leading to modern mammals and sauropsids [3]. As the genes encoding type 1 cysteine-rich keratins are the neighbors of a cluster of *KRTAP* genes in mammalian but not sauropsidian genomes [3], it is conceivable that the 3′-terminal truncation of a cysteine-rich keratin gene has generated the first *KRTAP* gene in mammals. Subsequently, this gene might have undergone duplications, mutations and translocations to generate the various subtypes of modern *KRTAP*s.

The results of our mRNA *in situ* hybridization experiments show that EDCRP is expressed in the subperiderm layer of embryonic epidermis prior to its cornification and shedding [27] as well as in the barbule cells of growing feathers prior to their cornification. Like KRTAP-mediated cornification of hair keratinocytes [5], EDCRP-mediated cornification of feather keratinocytes must be expected to abrogate the detectability of mRNAs *in situ*. Indeed, fully cornified subperidermal cells and feather cells that have already passed the EDCRP-positive differentiation stages do not yield *in situ* hybridization signals (Figure 2). The expression and cross-linking of EDCRP may contribute to the apparent stiffness of the subperiderm that allows its desquamation (together with the periderm) “in the form of extended epithelial sheets” [27]. The observed *in situ* hybridization pattern in feather follicles is compatible with the hypothesis that EDCRP is involved in the cysteine-dependent protein crosslinking and hardening of cells that become the building blocks of feathers.

Based on the detection of EDCRP expression in the subperiderm, a temporary embryonic skin layer, and in the feathers, a cyclically shed skin appendage, we put forward a model of feather development that emphasizes the constant topology of epidermal layers during the growth of feathers (Figure 3). This model integrates prior hypotheses about the link between the subperiderm and feather barbs and barbules [11,20], the key role of the tubular shape of the feather follicle in establishing the complex branching of feathers [19,28] and the role of cell death in removing cells that separate the branches of growing feathers [13,29,30]. In essence, a series of steps in embryonic skin development are replicated, in modified form, during feather growth and shedding in adult birds (Figure 3). Notably, the timing of EDCRP expression in feather follicles is decoupled from that in extra-follicular epidermis already in the embryo. To achieve completion of the complex morphogenesis of feathers before hatching, cell differentiation and expression of EDCRP (Figure 1B) are started in feather follicles at much earlier time points than in apteric (featherless) skin.

EDCRP is the second protein type, besides beta-keratins, which is expressed both in the subperiderm and in feathers [31]. The properties of feathers are likely to depend on both EDCRP and beta-keratins, which may interact via disulfide bonding or other mechanisms. However, EDCRP has a uniquely high content of cysteine and, different from beta-keratins, is present in birds but not in the phylogenetically closely related crocodilians. Therefore, EDCRP might have played a particularly important role in the evolution of feathers. The significance of our findings is further underscored by the finding of similarity of avian EDCRP to mammalian cysteine-rich KRTAPs, which indicates that the origin of highly cysteine-rich proteins was a key step in the evolution of both feathers and hair (Figure 7). Taken together, EDCRP appears to represent one of the critical innovations during the evolution of feathers which may be summarized as follows. (1) The evolutionary origin of the subperiderm in a common ancestor of archosaurs (crocodiles and birds as well as extinct dinosaurs) provided the cellular ancestors of cornified feather keratinocytes [11]. (2) The evolution of a feather follicle with tubular shape was an essential evolutionary innovation in the lineage leading to modern birds after its divergence from the crocodilian lineage [19]. (3) The origin of the *EDCRP* gene by duplication of an ancestral EDC gene [8] and/or the modification of its sequence to increase the cysteine content of EDCRP contributed to the ability of subperidermal keratinocytes to establish durable protein cross-links. It is likely that other cysteine-rich proteins evolved in parallel in birds. The extensive disulfide bonding facilitated the formation of delicate, yet stable structures of feathers. (4) The co-option of signaling and cell differentiation pathways facilitated the formation of the branching pattern of feathers. Dermo-epidermal interactions and differential cell growth and cell death processes in the adjacent layers of the feather follicle established the first feathers which gained complexity by the fine-tuning of epithelial growth and fusion processes during evolution [29,32].

Thus, it appears that the evolution of a structural protein complemented the evolution non-structural genes and regulatory elements [33,34]. Paleontological findings have unraveled a series of steps in the evolution of feather morphology [35]. Molecular biological studies, including the present characterization of EDCRP, should now help to elucidate the evolution of the feather architecture at the molecular level.

## Conclusion

In conclusion, this study suggests that the evolution of avian EDCRP has been instrumental in the evolution of feathers and that EDCRP contributes to the structural integrity of feathers in modern birds.

## Methods

### Comparative genomics and sequence analysis

The genome sequences from the bird species listed in Additional file 1: Table S1 [36] and from the following non-avian species were investigated: Chinese alligator (*Alligator sinensis*), American alligator (*American alligator*), saltwater crocodile (*Crocodylus porosus*), gharial (*Gavalis gangeticus*) [37], green sea turtle (*Chelonia mydas*), Chinese softshell turtle (*Pelodiscus sinensis*) [38], green anole lizard (*Anolis carolinensis*) [39], king cobra (*Ophiophagus hannah*) [40], Burmese python (*Python bivittatus*) [41], African clawed frog (*Xenopus laevis*), fugu (*Takifugu rubripes*), platypus (*Ornithorhynchus anatinus*), opossum (*Monodelphis domesticus*) and human (*Homo sapiens*). All genome sequences are available in the GenBank. The tBLASTn algorithm (http://www.ncbi.nlm.nih.gov/) was used to screen for homologs of chicken EDCRP [8]. Amino acid sequences of both complete EDCRP and distinct motifs of EDCRP were used as queries. Sequences were aligned using the programs MUSCLE and Multalin [42]. Genomic DNA of the ostrich was prepared from commercially marketed ostrich meat, amplified with the primers 5′-AGAAGTCCAGCCGCTGTGTCA-3′ and 5′-GGTATGCAGTACTTTCTCATGG-3′ and sequenced. Genomic DNA from a zebra finch (kindly provided by Dr. Lorenzo Alibardi, University of Bologna, Italy) was amplified with the primers 5′-TGCTCTGTCGTGAAGAGCAAG-3′ and 5′-CGGGCTTCTTCACCACGTAG-3′. The PCR product was purified and sequenced [GenBank: KP224277].

### RT-PCR and sequencing

RNA was prepared from chicken embryos on embryonic days 10, 14, 18 and from adult chicken as described previously [12]. All animal procedures were approved by the Animal Care and Use Committee of the Medical University of Vienna (66.016/0014-II/3b/2011). The RNAs were reverse-transcribed and subjected to PCRs with intron-spanning primer pairs specific for EDCRP (5′-CTCAACTGAACCCCTCAGTTAG-3′ and 5′-CAGCACACTGTCTTGCTCTTC-3′) and caspase-3, as a control (5′-TGGCGATGAAGGACTCTTCT-3′ and 5′-CTGGTCCACTGTCTGCTTCA-3′).

### mRNA *in situ* hybridization

A probe annealing to the 3′-untranslated region of chicken EDCRP mRNA (nucleotides 11–286 downstream of the stop codon) was cloned in sense and antisense orientation into pCR®2.1-TOPO® plasmids (Life Technologies, Paisley, UK) and transcribed *in vitro* using the DIG RNA labeling kit (Roche Applied Science). The *in situ* hybridizations with antisense and sense probes were performed at a hybridization temperature of 45°C (incubation time 1 h) on sections of formaldehyde-fixed and paraffin-embedded chicken tissues according to a published protocol [12].

## Additional files

Additional file 1: Table S1. Location of avian *EDCRP* genes and properties of encoded proteins. Additional file 2: Figure S1. Amino acid sequences of avian EDCRPs. Amino acid sequences were obtained by conceptual translation of the coding regions of *EDCRP* genes (Additional file 1: Table S1). Additional PCRs and sequencing reactions were performed on genomic DNA from *Taeniopygia guttata* and *Struthio camelus australis*. For the latter species, the sequencing results of our experiments were used to replace ambiguous parts of the gene sequences in the GenBank. Because of the incompleteness of the genome sequence, the amino acid sequence of EDCRP of *Tinamus guttatus* could be determined only partially; the unknown sequence is indicated by a series of “x”. The amino acid residues C, K, P and Q are highlighted with the same colors as in Figure 4. Additional file 3: Figure S2. Alignment of nucleotide sequences of the proximal promoter and exon 1 of *EDCRP* genes. The nucleotide sequences of the promoter and exon 1 of avian *EDCRP* genes were aligned. The transcription of exon 1 and mRNA splicing to exon 2 were confirmed in the chicken, as described previously [8]. Red fonts indicate positions with identical nucleotides in all species. TATA-like elements (AATAAA) are highlighted by yellow shading. Green shading marks the nucleotide change that converts this element into a canonical TATA box in the loon. The splice donor sites (GT) at the starts of intronic sequences are underlined. Species: budgerigar (*Melopsittacus undulatus*), chicken (*Gallus gallus*), duck (*Anas platyrhynchos*), falcon (*Falco cherrug*), flycatcher (*Ficedula albicollis*), loon (*Gavia stellata*), ostrich (*Struthio camelus australis),* pigeon (*Columba livia*)*,* tinamou (*Tinamus guttatus*), zebra finch (*Taeniopygia guttata*). Additional file 4: Figure S3. A canonical TATA box is conserved among the promoters of chicken and lizard EDC genes but mutated in the promoter of chicken *EDCRP*. Alignment of promoter and exon 1 nucleotide sequences of EDC genes of the chicken (Gg, *Gallus gallus*) and the lizard (Ac, *Anolis carolinensis*). The genes and their positions in the genome sequences have been reported previously [8]. Red fonts indicate nucleotides that are conserved in at least 85% of the sequences, and blue fonts indicate nucleotides that are conserved in at least 50% of the sequences. The consensus sequence is shown below the alignment. The position of TATA box, which has been replaced by a TATA-like element (AATAAA) in chicken EDCRP, is indicated. The splice donor sites (GT) at the starts of intronic sequences are underlined. Additional file 5: Figure S4. The number of EDCRP sequence repeat units varies among bird species. The numbers of central sequence EDCRP repeats (indicated in Figure 4) were mapped onto a phylogenetic tree of birds. Only sequence repeats containing at least the 6 first residues of the repeat unit, i.e. CCDPCQ or similar, were counted. *, chicken EDCRP has 3 additional incomplete repeat units. The exact number of repeat units of the tinamou is not known because of incompleteness of the gene sequence.

## Abbreviations

BLAST: Basic local alignment search tool
EDCRP: Epidermal differentiation cysteine-rich protein
EDC: Epidermal differentiation complex
KRTAP: keratin-associated protein
PCR: Polymerase chain reaction
RT: Reverse transcription
SEDC: Simple EDC gene
SPRR: Small proline-rich protein
