# Supplementary material for: Convergent evolution of cysteine-rich proteins in feathers and hair
Source: BMC Evol Biol. 2015 May 7;15:82. doi: 10.1186/s12862-015-0360-y (PMC4423139; doi:10.1186/s12862-015-0360-y)
Supplement: Additional file 1: Table S1. — Location of avian EDCRP genes and properties of encoded proteins. [file 12862_2015_360_MOESM1_ESM.pdf]

Table S1. Location of avian *EDCRP* genes and properties of encoded proteins

Species	Accession number of genome sequence	Exon 1 end	Exon 2 start	CDS start	CDS end	Length of encoded protein (number of amino acid residues)	Cysteine content (%)
Anas platyrhynchos	NW_004677042.1	5883	6352	6391	7227	278	34.9
Aptenodytes forsteri	NW_008794583.1	549076	548551	548512	548072	146	35.6
Columba livia	NW_004973834.1	152042	151578	151540	150731	269	33.8
Egretta garzetta	NW_009258273.1	91304	91861	91900	92748	282	35.1
Falco cherrug	NW_004994475.1	104715	104188	104153	102819	444	34.7
Ficedula albicollis	NW_004775766.1	55545	54730	54691	53579	370	37.6
Gallus gallus	NC_006112.2	775643	775187	775144	774512	385	36.4
Gavia stellata	NW_009321886.1	24720	25256	25295	26320	341	34.3
Geospiza fortis	NW_005054641.1	263276	262455	262416	260914	500	38.2
Mesitornis unicolor	NW_010170027.1	3726	4178	4217	4930	237	32.5
Pygoscelis adeliae	NW_008824590.1	97907	98440	98475	99188	237	35.9
Serinus canaria	NW_007931203.1	132963	132225	132186	131530	218	35.3
Struthio camelus australis	NW_009271250.1	29800	29449	29404	28640	228/247 (2 forms)	29.4/29.6
Taeniopygia guttata	NW_002198052.1	49588	48897	48858	47870	330	37.1
Tinamus guttatus	NW_010582719.1	58782	59108	59152	59922		
	NW_010628873.1	n.a.	n.a.	1	243	>338	30.5

Notes: CDS, coding sequence. *Struthio* : sequence gap between nucleotides 28818 and 29223; length of protein forms deduced from PCR product sequences. *Tinamus* : parts of the CDS are present on 2 different scaffolds. n.a., not applicable.
