# Supplementary material for: Convergent evolution of cysteine-rich proteins in feathers and hair
Source: BMC Evol Biol. 2015 May 7;15:82. doi: 10.1186/s12862-015-0360-y (PMC4423139; doi:10.1186/s12862-015-0360-y)
Supplement: Additional file 2: Figure S1. — Amino acid sequences of avian EDCRPs. Amino acid sequences were obtained by conceptual translation of the coding regions of EDCRP genes (Additional file 1: Table S1). Additional PCRs and sequencing reactions were performed on genomic DNA from Taeniopygia guttata and Struthio camelus australis. For the latter species, the sequencing results of our experiments were used to replace ambiguous parts of the gene sequences in the GenBank. Because of the incompleteness of the genome sequence, the amino acid sequence of EDCRP of Tinamus guttatus could be determined only partially; the unknown sequence is indicated by a series of “x”. The amino acid residues C, K, P and Q are highlighted with the same colors as in Figure 4. [file 12862_2015_360_MOESM2_ESM.pdf]

>Anas_platyrhynchos_EDCRP
MCHSGCCSTGCCSVVKSKTVCCSPCQKTVCCDPCQQSCCDPCQQSCCDPCQKTVCCSPCQQSCCDPCQQSCCDPCQQSCCDPC
QQSICCDPCQKPCCDPCQQSICCDPCQKPPCDPCQQSCCDPCQAVCCDPCQQSICCDPCQKPCCDPCQQSCCHPCQQSCCHPC
QQSCCDPCQQSCCDPCQQSCCDPCQKPCCDPCQQSCCDPCQKTCCCCCGQQCVRTCCCGCRPCCCSGCLSCCSYVVKKKPVVV
CCSPVNYCSPMRKYCIPIQQCCTTIKKCC

>Aptenodytes_forsteri_EDCRP
MCSTRCCSTGCCSVVKSKTVCCSQPCQKTVCCDPCQQSCCCDPCQKPCCDPCQQSCCDPCKPCCDPCQQSVCCTKVCQKSCCC
CSQRPCCCCGCCPCCCPGCLSCCSYVVKKKPAVVCCSPVRYCSPMRKCCIPIQQCCTTVKKCC

>Columba_livia_EDCRP
MCSTGCCPIVKSKTVCCSQPCQSVCCDPCQKKTVCCSPCQQSICCDPCQQSCCDPCQKRCCDPCQKQCCDPCQQSCCDPCQKQ
CCDPCQQSCCDPCQKQCCDPCQQSCCDPCQKTCCDPCQQSCCDPCQQSCCNPCQQSCCNPCQQSCCNPCQQSCCDPCQQSSCC
DPCQQSCCDPCQQSSCCDPCQQSCCDPCQRSVCCTKVCQQSCCVQPCCRCGCCPCCCSGRLSCCSYMVKKPVVVCCSPVQYCS
PLRKCSIPIQQCCASIKKSC

>Egretta_garzetta_EDCRP
MCSSGCCSTGCCSVVRSKTVCCSQPCQQTICCDPCQKSVCCSPCQQSRCCDPCQKPCCDPCQQSCCDPCQQSCCDPCQKPCCD
PCQQSCCDPCQQSCCDPCQKPCCDPCQQSCCDPCQQSCCDPCQKPCCDPCQQSCCDPCQKPCCDPCQQSCCDPCQKPCCDPCQ
QSCCDPCQRSCCDPCQKPCCDPCQQSCCDPCQKPCCNPCQKSVCCTKVCQKSCCCCGQRPCCCCGCHPCCCSGCLSSCSYVVK
KKPVVVCYSPCCSPARKCCIPIQQCCTTIKKGC

>Falco_cherrug_EDCRP
MCSTGCCSTGCCSIVKSKTVCCSQPCQKTICCEPCQTSVCCSPCQQSCCCDPCQKPCCDPCQKPCCDPCQTTVCCDPCQKPCC
DPCQKPCCDPCQQSVCCDPCQKPCCDPCQQSVCCDPCQKPCCDPCQQSVCCDPCQKPCCDPCQQSVCCDPCQKPCCDPCQQSV
CCDPCQKPCCDPCQQSVCCDPCQKPCCDPCQQSVCCDPCQKPCCDPCQQSVCCDPCQKPCCDPCQQSVCCDPCQKPCCDPCQQ
SVCCDPCQKPCCDPCQQSVCCDPCQKPCCDPCQQSVCCDPCQKPCCDPCQQSVCCDPCQKPCCDPCQQSVCCDPCQKPCCDPC
QQSCCDPCQKPCCDPCQQSVCCDPCQQSCCDPCQQPVCCTKVCQPSVCCCGQRPCCCCGCRPCCCSGCQSCCSYVVKKKPVMV
CCSPMQCCSPMRKYCIPIQQCCAAIKKRC

>Ficedula_albicollis_EDCRP
MCSTGCCSTGCCSVVKSKTVCCTPCKTVCCSPCKTVCCSPCKTVCCSPCKTVCCSPCQQSCCCDPCQKTVCCDPCQSCCDPCQ
SCCDPCQKPCCDPCQSCCDPCQSCCDPCQKPCCDPCQSCCDPCQSCCDPCQKPCCDPCQSCCDPCQSCCDPCQKPCCDPCQSC
CDPCQSCCDPCQKPCCDPCTVCCDPCQKPCCDPCQKPCCGPCQTVCCDPCQKPCCDPCQSCCDPCQSCCDPCQKPCCDPCQSC
CDPCQSCCDPCQKPCCDPCQSCCDPCQKPCCDPCQSCCDPCQKPCCDPCQSCCDPCQQSVCCTKVCQKSVCCVPRPCCCPCGS
LSCCSCVVKKPVVVCCTPVRRFCTPCIPIQQCCIKKSC

>Gallus_gallus_EDCRP
MCPNVCCSSGCCSVVKSKTVCCSPCQKTVCCDPCQKTVCCEKTVCCDPCQKPCCDPCQTCCDPCQKPCCDPCQTCCDPCQKPC
CDPCQTCCDPCQTCCDPCQKPCCDPCQQSVCCDPCQKPCCDPCQTCCDPCQKPCCDPCQTCCDPCQKTVCCDPCQTCCDPCQK
TVCCDPCDPCQTCCDPCQKPCCDPCQTCCDPCQKPCCDPCQTCCDPCQKTVCCDPCQKPCCDPCQTCCDPCQKTVCCDPCQKP
CCDPCQTCCDPCQTCCDPCQKTVCCDPCQKTVCCDPCQKPCCDPCQTCCDPCQKPCCDPCQTCCDPCQKPCCSCCSQQCLQTC
CRPCCCSGRLSCCSYVVKKKPVVVCCSPVQYCSPLRKCCVPIRQCCTTVKKCC

>Gavia_stellata_EDCRP
MCSSGCCSTGCCSVVKSKTVCCSQPCQRTICCDPCQKTVCCSPCQQSCCCDPCQQSICCDPCQKPCCDPCCCDPCQQSICCDP
CQKPCCDPCQQSICCDPCQQSCCDPCQKPCCDPCQQSVCCDPCQQSCCDPCQKPCCDPCQQSVCCDPCQQSCCDPCQKPCCDP
CQQSVCCDPCQQSCCDPCQKPCCDPCQQSCCDPCQKPCCDPCQQSCCDPCQKPCCDPCQQSCCDPCQKPCCDPCQQSCCDPCQ
KPCCDPCQQSCCDPCQKPCCDPCQQSCCDPCQKPCCDPCQHSSCCSGCLSCCSYVVKKKPVMVCCSPVQYCSPMRKYCIPIQQ
CCTTIKKGC

>Geospiza_fortis_EDCRP
MCSTGCCSTGCCSVVKSKTVCCTPCKTVCCSPCKTVCCSPCKTVCCSPCKTVCCSPCKTVCCSPCKTVCCSPCQQ
SCCCDPCQKPCCDPCQSCCDPCQSCCDPCQKPCCDPCQTCCDPCQKPCCDPCQTCCDPCQKPCCDPCQTCCDPCQ
KPCCDPCQTCCDPCQKPCCDPCQTCCDPCQKPCCDPCQTCCDPCQKPCCDPCQTCCDPCQKPCCDPCQTCCDPCQ
KPCCDPCQSCCDPCQKPCCDPCQSCCDPCQSCCDPCQKPCCEPCKTVCCDPCQTCCDPCQKPCCDPCQSCCDPCQ
KPCCDPCQSCCDPCQKPCCDPCQTCCDPCQKPCCDPCQTCCDPCQKPCCDPCQSCCDPCQKPCCDPCQTCCDPCQ
KPCCDPCQSCCDPCQKPCCDPCQSCCDPCQKPCCDPCQSCCDPCQSCCDPCQKPCCDPCQTCCDPCQSVCCTKVC
RKSVCCVPRPCCSPCCVVKKPVVVCCTPVRKCCTPCIPIQQCCASLRKTC

>Mesitornis_unicolor_EDCRP
MCSTGCCSVVKSKTVCCSQPCQKIICCDPCQRSVCCDPCQQSICCDPCQKPCCDPCQQSCCDPCQQSCDPCQQSCCDPCQQSC
CDPCQQSCDPCQQSCDPCQQSCCDPCQQSCCDPCQKPCCDPCQQSCCDPCQQSCCDPCQQSCCDPCQQSCCNPCQQTVCCTKV
CQTPCCAQQCLQPCCCSCRPCCSGGVSCCTYAVKKKPAVVCCSPVQCCSPCPPRKCSIPIQQCCAAIKKRC

Figure S1 (continued)

>Pygoscelis_adeliae_EDCRP
MCSTRCCSTGCCSIVKSKTVCCSQPCQKTVCCDPCQQSCCCDPCQKPCCDPCQQSVCCDPCQKPCCDPCQQSVCCDPCQQSCC
DPCQKPCCDPCQQSCCDPCQKPCCDPCQQSCCDPCQKPCCDPCQQSCCDPCQKPCCDPCQQSCCDPCQKPCCDPCQQSVCCTK
VCQKSCCCCSQRPCCCCGCCPCCCPGCLSCCSYVVKKKPVVVCCSPVRYCSPMRKCCTPIQQCCTTIKKCC

>Serinus_canaria_EDCRP
MCSTGCCSTGCCSVVKSKTVCCTPCKTVCCTPCKTVCCSPCKTVCCSPCKTVCCSPCKTVCCSPCQQSCCCDPCKKTVCCEPC
QSCCDPCQKPCCDPCQSCCDPCQSCCDPCQKPCCDPCQSCCDPCQKPCCDPCQTCCDPCQKPCCDPCQSCCDPCQSVCCTKVC
HKSVCCVPRPCCSPCCSCVVKKPVVVCCTPVRKYCTPCIPIQQCCASLRKTC

>Struthio_camelus_australis_EDCRP_form_1
MCSSGKTRTVCCSQPCQQSSCCDPCQKSSRCVKPCQQSSCCDPCQQSSCCDKPCQQSSCCDPCQQSSCCDQPCQQSSCCDPCQ
QSSCCDQPCQQSSCCDPCQQSSCCDQPCQQSSCCDPCQQSSCCDKPCQQSSCCDLCQQSTCCDPCQQSTCCVKPCKKSSYCCT
QRCLPCSRCGCISCCCGSVSCCSTVVKKKPVVVCCSPLCYCSPMRKYCIPTQQCCYSVKKCY

>Struthio_camelus_australis_EDCRP_form_2
MCSSGKTRTVCCSQPCQQSSCCDPCQKSSRCVKPCQQSSCCDPCQQSSCCDKPCQQSSCCDPCQQSSCCDQPCQQSSCCDPCQ
QSSCCDKPCQQSSCCDPCQQSSCCDQPCQQSSCCDPCQQSSCCDQPCQQSSCCDPCQQSSCCDKPCQQSSCCDLCQQSTCCDP
CQQSTCCVKPCKKSSYCCTQRCLPCSRCGCISCCCGSVSCCSTVVKKKPVVVCCSPLCYCSPMRKYCIPTQQCCYSVKKCY

>Taeniopygia_guttata_EDCRP
MCSTGCCSTGCCSVVKSKTVCCSPCKTVCCSPCKTVCCSPCKTVCCSPCKTVCCSPCQQSCCCDPCQKTVCCCDPCQSCCDPC
QKPCCDPCQTCCDPCQKPCCDPCQTCCDPCQKPCCDPCQTVCCDPCQKPCCDPCQTCCDPCQKPCCDPCQTCCDPCQKPCCDP
CQTCCDPCQKPCCDPCQTVCCDPCQKPCCEPCQTCCDPCQKPCCEPCQTCCDPCQSCCDPCQKPCCDPCQTCCDPCQTCCDPC
QKPCCDPCQTCCNPCQSVCCTKVCQKSVCCVPRPCCCPCGSQSCCSYVVKKPVVVCCTPVRKCCTPCIPIQQCCASLRKTC

>Tinamus_guttatus_EDCRP_partial
MCSRGKTVCCSQPCQQARCCDPCQQATCCDPCQQARCCVKVCQKSTCCDPCQQSSCCDPCQQSSCNPCQQSSCCDPCQKPTCC
DPCQQSTCCDPCQKSVCCDPCQKPTCCDPCQQSTCCDPCQQSTRCDPCQKSVCCDPCQKSVCCDPCQQPTCCDPCQKSTCCDP
CQQSTCCDPCQQSTCCDPCQKSVCCDPCQKPTCCDPCQQSTCCDPCQQSTCCDPCQQTSCCNPCQQPICCTQPCTQRCLPCSR
CGWFPRCCxxxxxxxxxxCQQTSCCNPCQQPICCTQPCTQRCLPCSRCGCFPCCCGALSCCSAVVKRPVVVSCSPVSY
CSPLSKYCVPTQQSCYPIRRC

Figure S1. Amino acid sequences of avian EDCRPs. Amino acid sequences were obtained by conceptual translation of the coding regions of *EDCRP* genes (Additional file 1: Table S1). Additional PCRs and sequencing reactions were performed on genomic DNA from *Taeniopygia guttata* and *Struthio camelus australis*. For the latter species, the sequencing results of our experiments were used to replace ambiguous parts of the gene sequences in the GenBank. Because of the incompleteness of the genome sequence, the amino acid sequence of EDCRP of *Tinamus guttatus* could be determined only partially; the unknown sequence is indicated by a series of "x". The amino acid residues C, K, P and Q are highlighted with the same colors as in Figure 4.
