# Supplementary material for: Convergent evolution of cysteine-rich proteins in feathers and hair
Source: BMC Evol Biol. 2015 May 7;15:82. doi: 10.1186/s12862-015-0360-y (PMC4423139; doi:10.1186/s12862-015-0360-y)
Supplement: Additional file 3: Figure S2. — Alignment of nucleotide sequences of the proximal promoter and exon 1 of EDCRP genes. The nucleotide sequences of the promoter and exon 1 of avian EDCRP genes were aligned. The transcription of exon 1 and mRNA splicing to exon 2 were confirmed in the chicken, as described previously [8]. Red fonts indicate positions with identical nucleotides in all species. TATA-like elements (AATAAA) are highlighted by yellow shading. Green shading marks the nucleotide change that converts this element into a canonical TATA box in the loon. The splice donor sites (GT) at the starts of intronic sequences are underlined. Species: budgerigar (Melopsittacus undulatus), chicken (Gallus gallus), duck (Anas platyrhynchos), falcon (Falco cherrug), flycatcher (Ficedula albicollis), loon (Gavia stellata), ostrich (Struthio camelus australis), pigeon (Columba livia), tinamou (Tinamus guttatus), zebra finch (Taeniopygia guttata). [file 12862_2015_360_MOESM3_ESM.pdf]

```
    chicken  TTAAAAAATTGTAAAAAATTCACAGAATAAATTGATAATAACACAAAATTGAGGGGTTGGTCAAAAATAATGAGGCGAAGACCAAACAGA
       duck  TTAAAAAATTGTAAAAAATTTACTGAACAAATTGATAATAATACAAAATTGAGGG-TTGGTCAAAAATAATGAGGCAAAGACTAAATAGA
     pigeon  TTAAAAAATTGTAAAAAATTTACTGAACAAATTGATAATTACACAAAATTGAGGGACTGGTCAAAAGCAATGAGACAAAAACTAAATAGA
       loon  TTAAAAAATTGTAAAAAATTTACAGAACAAATTGATAATAACACAAAATTGAGGGGTTGGTCAAAAACAATGAGGCAAAGACTACACAGA
     falcon  TTAAAAAATTGTAAAAAATTTACAGAACAGATTGATAATGACACAAAATTGAGGGGCTGGTCAAAAACCACGAGGCAAAGACTAAATAGA
 budgerigar  TTAAAAAACTGTAAAAAATTTACAGAACAAATGGATAATAACTCAGAATGGATGGGTTGGTCGGAAATAATGAGGCAAAGACTAAACAGA
 flycatcher  TTAAAAAATTGTAAAAAATTCACACGACAAATTGGTAATGACCCAAAATTGAGGGGTTGGTCAAAAGCCACGAGGCAAAGACTAAACACA
zebra finch  TTAAAAAATTGTAAAAAATTCACACGACAGATTGATAATAACCCAAAATTGAGGGGTTGGTCAAAAGCCACGAGGCAAAGACTGAACACA
    ostrich  TTAAAAAACTGTAAAAAATTTACAGAACAAATTGGTAATAATACAAAATTGAGGGGGTGGTCAAAAATAATGAGGCAAATACTAAGTAGA
    tinamou  TTAAAAAACTGTAAAAAATCTGCAGAACAAATTGTTAATAATACAAAATTGAGGGGGTGGTCAAAAATAATGAGGCAGAGGCTAACCAGA

    chicken  TGCGGGCACATTCAACAAATAAGTATAGGCTTAGTGGGTAAATCACAA-------ATTGCTGTGGGGTACCTCATTTCCGTCAGGGCCAT
       duck  TGAAGGCACATTCAACAAATAAGTATAGGCTTAGTGGGTAAATCACGA-------ATTGCTTTGGGGTATCTCATTTCCGTCAGGGCCAT
     pigeon  TGAAGGCCTATTCAACAAGTAAGTATAGGCTTAGTGCGTAAATCACAA-------ATTGCTGTGGGGTACCTCATTTCCGGCAGGACCAT
       loon  TGAAGGCACATTCAACAAATAAGTATAGGCTTAGTGCGTAAATCACAA-------ATTGCTGTGGGGTACTTCATTTCCGGCAGGACAAT
     falcon  TGAAGGGACATTCAACAAGTAAGTATAGGCTTAGTGCGTAAATCACAA-------ATTGCTGTGGGGTACCTCATTTCCGGCAGGACAAT
 budgerigar  TGAAGGCCCATTCAACAAATAAGTATAGGCTTAGCACATAAATCACGA-------ATTGCTGTGGGGTACCTCATTTCCGGAAGGACAAT
 flycatcher  TGAAGGCCCATGGGGTGAATAAGAACAGGCTTGGTGCGTAAATCACGG-------ATTGCTGGGGGGTACCTCATGTCCGGCAGGTCAAT
zebra finch  TGAAGGCCCATTGGGTGAATAAGTACAGGCTTAGTGCGTAAATCACGG-------ATTGCTGGGGGGTACCTCATGTCCGGCAGGACAAC
    ostrich  TGAGGGCACATTCAACAAATAAGTATAGGTTTAGTGGGTAAATGATGAAGTCAGAATTGCTATGGGGTATCTCATTTCCGGCAGGCAGAT
    tinamou  TGAGGGCACGTTCAACGAATAAGTATAGGTTAGGTGGGCAAATGATGAAGTCAGAATTGCTATGGGGTATCTCATTTCCGACAGGCAAAT

                       TATA-like                                  exon1                      intron
    chicken  TTTCAGCTAAATAAAAGGTCTCCCCATCCCTTTGCTCCTCACTCAACTGAACCCCTCAGTTAGCAAGGTAAGTGTTACCTAGT-GTTGTC
       duck  GTCGAGCTAAATAAAAGGTCTCCCCGTCCCTTTGCTCCTCACTCAACTGAACCACTCAGTTAGCAAGGTAAGTGTTACCTAGT-GCTGTT
     pigeon  TTCGAGCTAAATAAAAGGTCTCCCTGTCCCTTTGCTCCTCACTCAACTGCACCTCTCAGTCAGCGCGGTAAGTGTGACCCATTAGCAGCG
       loon  TTCGAGCTATATAAAAGGTCTCCCTGTCCCTTTGCTCCTCACTCAACTGCACCACTCAGTTAGCACGGTAAGTGTTACCTATTAGCAGTG
     falcon  TTTGAGCTAAATAAAAGGTCTCCCCATCCCTTTGCTCCTCACTCAACTGCACTGCTCAGCTAGCACGGTAAGTGTTAGCTATTAGCAGTG
 budgerigar  TTGGAGCTAAATAAAAGGTCTCCCCATCCCTTTGCTCCTCACTCAACTGCACCACTCAGTTAGCACGGTAAGTGTTACCCATTAGCGGTG
 flycatcher  CCTGACCTCAATAAAAGCTCCCCGGGTGCCTCTGCTCCTCACACACCTTCAGCTCCCAGCAAGAACGGTGAGTGTCTCCTGCCAGGGATC
zebra finch  TCCGACCTGAATAAAAGGTCTCCAGGTGCCTCTGCTCCTCACACACCTTCAGCTCTCAGCAAGAACGGTGAGTGTTTCCTACCAGGGATC
    ostrich  TTCGAGCTAAATAAAAGGCTTCCC-ATCCTTTTGCTCCTCACTCAACTACACCGCTCCATTAACACGGTAAGTCTCACCTGCTGGCACTG
    tinamou  TTCGAGCTAAATAAAAGGCCTCCC-ATCCTTTTGCTCCTCACTCAACTACACCACCCCGTTAGCACGGTAAGT-TCACCTACTGGCAACG
```

Figure S2. Alignment of nucleotide sequences of the proximal promoter and exon 1 of *EDCRP* genes. The nucleotide sequences of the promoter and exon 1 of avian *EDCRP* genes were aligned. The transcription of exon 1 and mRNA splicing to exon 2 were confirmed in the chicken, as described previously [8]. Red fonts indicate positions with identical nucleotides in all species. TATA-like elements (AATAAA) are highlighted by yellow shading. Green shading marks the nucleotide change that converts this element into a canonical TATA box in the loon. The splice donor sites (GT) at the starts of intronic sequences are underlined. Species: budgerigar (*Melopsittacus undulatus*), chicken (*Gallus gallus*), duck (*Anas platyrhynchos*), falcon (*Falco cherrug*), flycatcher (*Ficedula albicollis*), loon (*Gavia stellata*), ostrich (*Struthio camelus australis*), pigeon (*Columba livia*), tinamou (*Tinamus guttatus*), zebra finch (*Taeniopygia guttata*).
