# Supplementary material for: Convergent evolution of cysteine-rich proteins in feathers and hair
Source: BMC Evol Biol. 2015 May 7;15:82. doi: 10.1186/s12862-015-0360-y (PMC4423139; doi:10.1186/s12862-015-0360-y)
Supplement: Additional file 4: Figure S3. — A canonical TATA box is conserved among the promoters of chicken and lizard EDC genes but mutated in the promoter of chicken EDCRP. Alignment of promoter and exon 1 nucleotide sequences of EDC genes of the chicken (Gg, Gallus gallus) and the lizard (Ac, Anolis carolinensis). The genes and their positions in the genome sequences have been reported previously [8]. Red fonts indicate nucleotides that are conserved in at least 85% of the sequences, and blue fonts indicate nucleotides that are conserved in at least 50% of the sequences. The consensus sequence is shown below the alignment. The position of TATA box, which has been replaced by a TATA-like element (AATAAA) in chicken EDCRP, is indicated. The splice donor sites (GT) at the starts of intronic sequences are underlined. [file 12862_2015_360_MOESM4_ESM.pdf]

```
                  TATA                                                                                                    intron
Gg_EDCRP   TTTTCAGCTAAATAAAAGGTCTCCC-------CATC--------------------CC-----TTTGCTCCTCACTCAACTGAACCCCTCAGTTAGCAAGGTAAGTGTTAC
Gg_EDMPN   GCGAAGGGACTATAAAAGGTCTCAG-------GGTCCAGGGCACTTCATTC-GCTCCT-----CTCCTCTTCTCTCCACTTCTCCTCACCGTTCAGCAGGGTAAGTGAGAG
Gg_EDGH    CTAAACGCCATATAAAAGTTCCTGTGTCT----TAAAGCTCTTCATTCAGTCAACTT--------TAATCTTCTAAATGTGTTTCCAATGGCTGAGGAAGGTAAGTTCGCT
Gg_EDYM1   TCAGAGACCGTATAAAAACACACAG-------CTCCGCA-----------------CC---------TCCAATCCAGTTCTTACTCTCCTCTCTGAGCTCGGTAAGTTTTTT
Gg_EDYM2   TTATTCACTATATAAAAGCTCAGCC-------ATCACGGGGCT----------CTCCT-----AGGCACTTCTCTCCGGTTGTTCTCACTGATGAAGTCGGTAAGTCTGGC
Gg_EDMTF1  ACTGTTGCCCTATAAAAGCCCGCAC-------TGCTGACAGCTCT-----------GC-----ATTCACTTCTCCTGCCTTAGCTCCTT-GCACAAGTGGGTAAGTCAGAA
Gg_EDMTF2  CAGATACATATATAAAAGGGGGAGA-------AATCCAGGGCTCT-----------CT-----ACTCGCACCTCCCGGCTTACGTTCTC-GCTGAGCTTGGTAAGTGGGAC
Gg_EDMTF3  CAGATACATATATAAAAGGGGGAGA-------AATCCAGGGCTCT-----------CT-----ACTCGCACCTCCCGGCTTACGTTCTC-GCTGAGCTTGGTAAGTGGGAC
Gg_EDMTF4  AAAGAAACAGTATAAAAAGGGCTCTGAGCTCC---AC-GCTCTCCATCA----GCTC---------------TCTCACCTTCCTCCTCCTGGTGAACTCGGTAAGCCTTCA
Gg_EDbeta  TAAAAGCGAGTATAAAAGCACTCAC-------CATCAGG-----------------CT-----GTGTCTGACCATTTCTCTTATTTCTGTTGCGCAGTAGGTAAGCTTGAT
Gg_LOR1    CTTGGAAGGGTATAAAACTGAAGCACA---TCGGGTTTCCTCACTCCAT-----------TGGACTTCCTT----CAGCTTGTTCGTGCCGTGGGACACGGTAAGGCACCT
Gg_LOR2    CTAGAGGCAGTATAAAAGTTGCTTGGTTC----TGTAGTCCATCACTCGCTCAGAGT--------TGCGCTGTTGAACGTGTTATCAGAACCA-AGAAGGGTAAGTCAATT
Gg_LOR3    ACGAGCCCAGTATAAAGGCTAACGTATCC----TACGGCTCTTCATTCGCCTACCAC--------CAAGCCTTGCACAC----ATCCAGCTGTGTTGAAGGTAAGCCCATT
Gg_EDWM    TTTGTAACCCTATAAAAGGGCTCCG---------TCCCTTTGCCTCTCACAATCTGCT-------CCCCTGGTCTGCGTCTACCTTCCCTCATCACTTGGGTAAGGCAGCT
Gg_EDQM1   TGGGGACCTCTATAAAACTGCCCAT-------GTTCTGGAGGTTGTCAT-----TCCT-----TCCTCTCGCCTTCTTCTCA-----------AGCTTGGGTAAGTCTAAC
Gg_EDQM2   GGGTAAAGGGTATAAAAGCACTGTG-------GTCCCAGAGAGCCTCAT-----CGTC-----TCCGCATCCCTTCATTGCAGAGCA--------GCGCGGTAAGAGCAGT
Gg_EDQM3   CGAGGAACTATATAAAAGTTCCCAA-------ATGGGAGAGGAGCTCAT-----TCAC-----TCGGCTCCGTTCCCTCTGAGCAACCTTTCCAGACAAGGTGAGTGTTTG
Gg_EDCH1   TTTGGAGGGCTATAAAACCCA-CTCAGCCCAGTCCTCTCCACTCACCATCC--CTCCCAGACACTCC---TCTTGTTGCTGCAG--------------AGGTAAGGCTGCT
Gg_EDCH2   TTCGGAGCTGCATAAAAGCCTGCTCAGCCCAGTCCTCCTCACTTGCCATCC--CTTCCTGACTCTTC---TCCTGTTGCTGCAG--------------AGGTAAGGATGCT
Gg_EDCH3   ATTGGGAGCATATAAAAGTTCGTAT---------CCCTTGCTGCTCCTTCATTCAGCT-------TCACTTCTCAGTGTTCTTCTCTGCTCCTACAAAGGGTAAGTTGGAT
Gg_EDCH4   TTTGGAAGAGTATAAAAACTCCCTCACTCTGGTTTCCCTCATTCGCTCCTCTGCTCCCATACACATCCATTCCTGTTGCTGAAA--------------GGGTGAGTCTACT
Gg_EDCH5   CACGGCTGGATATAAAAGCTTTTGGCTCCAGTGTGGTCTTCAGGACTCGGACACTGC--------TCAGCCATCCACCAGCAGCCAC----------AGGGTGAGTTGGAT
Gg_EDQrep  TTAGCAGCTATATAAAAGGTCAGCT-------TCCATTAGGGC----------TCACT-----GGTCACATCACAACGCTGTTCTCATCGGGGAAGCTGGGTAAGTCTGCT
Gg_EDPE    TTTGAAACCATATAAAAGTTCCCAA-------ATCTCCACGCTGCTCCA-----TCCA-----CTCGA---CTTTGCACTTCTCCCCGTTGACTGGTTGGGTAAGTCACTG
Gg_EDDM    ATTGAAATTGTATAAAAGGGTTCATATTGTA--------GTGTTTCCCA----AGCCA-----TCTTGGTTTCGTTAGCTTGTTCCTGGTGGTGAATCGGGTAAGTCTCTC
Gg_EDSC    CCAGGCTCCCTATAAAAGACCTCAT-------AACCATTTCTCCTCATTCTCTCATCT-------TCACATCTCCCCTTGAGCTCTCACTGAACA---GAGTAAGTCCGGC
Ac_LOR     CTGAAGACAGTATAAAAAGATCTCTGAAGACA---AAAGCTCTTCATCC----GCTTA-----TGTGCAACTTCTCCTCTGGTTGCAGCTGCTGAAGAAGGTAAGCCAAAC
Ac_EDSC    GGTAAGATGGTATAAAAATCTCTTCGTCTCAAG--------CACTGCTCATACCTTCAGCGTCGCTTGACTTGGCTTGACCAAAGCTTCTCAGTGAACAAGTGAGTATGAG
Ac_EDCP    ATAAGAACTATATAAAAGAGCTGGT-------GTTCTTTTCTTCCTCAT-----CTAC-----TTGATCCTTGGTCCATTTGATCCC--------TTTTGGTAAGTCTGAT
Ac_EDEPT   CCACCTTCCATATAAAACCCAGCTCTCGGCGCAACACGCCTCTCCGCCTTGCGCTGTA-----GCATCATTTCTTCCCGGCACCAAGAAAGTTG-----GGTAAGCTCCAT
Ac_EDSPR1  AACAAAACTGTATAAAAAGAGGTGGCTTGTT--------GTCATTCCCA----ATCGT-----GCTTCGCTTTGG---------CTTGGCGTTGAGTCAGGTGAGTCCTGA
Ac_EDSPR2  TAAGAAATGGTATAAAAAACCACCGAAAGCTCCTGGCTCATCACTTCATCTGGTGGACTTTGCTCTTCACT----CAGCTCCGTCGTGTGCATTTGGGTGGTAAGGCTCTT
Ac_EDPQ2   GAGGACACTTTATAAATAGTCTATCCCTTCAAAGGGCCTGCATA--CTAATTTCTGCA------TATCCTCATCCGAGACGCAGCTCTTCCTTCCTGCAGGTAATCATATG
Ac_EDPQ3   TCGGGTGGCATATAAATCACTCCTCATCTCCCCAGGCTCCTCACTCCGCTTTGCTGCAACGCACCGTGTTCAGTCTTGATTCTGCCTTCCTT-----GTGGTAAGTAGACT
Gg_EDQCM   ACTCACGCCATATAAAAAGGTTTGCATTTTGCTGC----------CCGCATCAGTTCA------TTTGTCTACTCCCTCACTCACTTGTGCTGGTGAACGGTAAGTCTTTC
Ac_EDSQ    TGGGAAGCCATATAAAAGAGACGACAGGTCGGGGTGTCCTCCAAACCCAAACTGATCT------CTTGGATCTTCCAGCTGTTGTCAGCCACTGCAGTCGGTAAGTATCTG
Ac_EDSCP   GAGCATTCTGTATAAAAGGTCTCCC-------ATCGCCTGACTATTCCACCAGCTTCA-----TTCCCCTTCTCTTGACTTGTCTTCTCCAGCACTCTCGGTAAGTTGGAT
Ac_EDCM    GATGATGCCCTATAAAAGGGCGC----------AGAGGCTTCCCATCCATACACTGAA-----GCATCAGTTGCTTACGAACTTCCTACACTTGAGCAAGGTAAGGCTGAT
Ac_EDCQ1   AAGGAAACCATATAAAAGTCCTCAC--------TGTTCGCTCTGATAGA-----TACA-----CACCTGAGCTCTTGACTGATACACATTGACCTTTTGGGTAAGTTTGCA
Ac_EDCQ2   AAGGAGACTATATAAAAAGACCTCAGATCACACCCAAAGGCATACACCA----GCAG------TCTCTGCAATCGCACACTTCTCAGGCAGGACTTGGGGGTAAGTCGGAA
Ac_EDCQ3   AACCAAGCAGTATATAAGGTTCTCCTGTGTTGGACCTTTAGTCACACCCTTGGATCCA-----TCAGAGTTACCAGCTCCCACCACTGAATC-------GGTAAGTTGAAT
Ac_EDCRP   AAAAGCGGTATATAAAGGTGTCCG--TCC----TTCCACACTTCATTCAGTCCTCCA--------TTGTCTTACTACCACTTACAC---------GCAAGGTAAGAGCAAT
Consensus  ...gaa.c..TATAAAAg..c.c...................tc..c.........c........t....t.t.....ct...................GGTAAGtc...t
```

**Figure S3. A canonical TATA box is conserved among the promoters of chicken and lizard EDC genes but mutated in the promoter of chicken *EDCRP*.** Alignment of promoter and exon 1 nucleotide sequences of EDC genes of the chicken (Gg, *Gallus gallus*) and the lizard (Ac, *Anolis carolinensis*). The genes and their positions in the genome sequences have been reported previously [8]. Red fonts indicate nucleotides that are conserved in at least 85% of the sequences, and blue fonts indicate nucleotides that are conserved in at least 50% of the sequences. The consensus sequence is shown below the alignment. The position of the TATA box, which has been replaced by a TATA-like element (AATAAA) in chicken EDCRP, is indicated. The splice donor sites (GT) at the starts of intronic sequences are underlined.
