# Supplementary material for: Convergent evolution of cysteine-rich proteins in feathers and hair
Source: BMC Evol Biol. 2015 May 7;15:82. doi: 10.1186/s12862-015-0360-y (PMC4423139; doi:10.1186/s12862-015-0360-y)
Supplement: Additional file 5: Figure S4. — The number of EDCRP sequence repeat units varies among bird species. The numbers of central sequence EDCRP repeats (indicated in Figure 4) were mapped onto a phylogenetic tree of birds. Only sequence repeats containing at least the 6 first residues of the repeat unit, i.e. CCDPCQ or similar, were counted. *, chicken EDCRP has 3 additional incomplete repeat units. The exact number of repeat units of the tinamou is not known because of incompleteness of the gene sequence. [file 12862_2015_360_MOESM5_ESM.pdf]

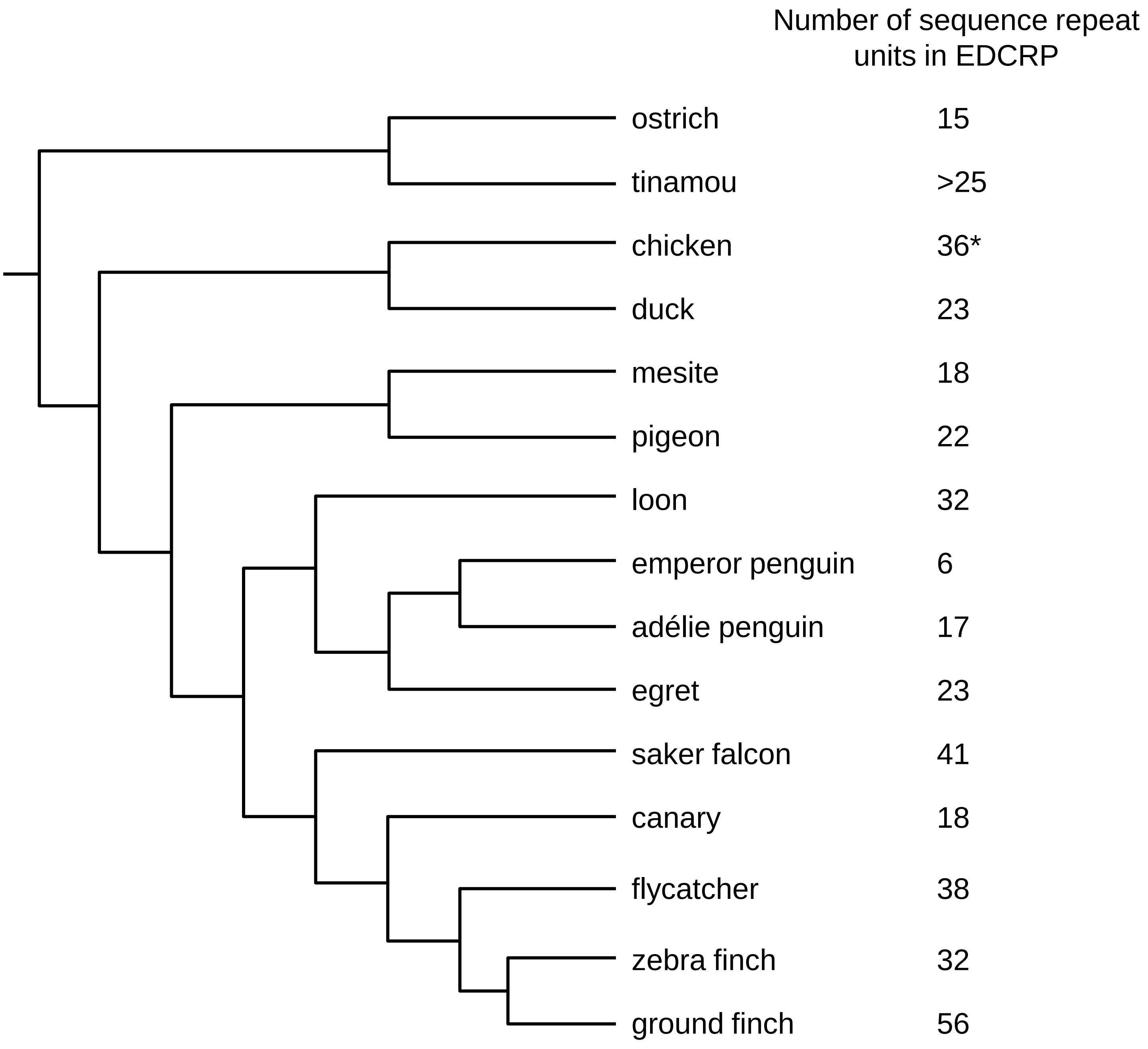

Figure S4. The number of EDCRP sequence repeat units varies among bird species. The numbers of central sequence repeats (indicated in Figure 4) were mapped onto a phylogenetic tree of birds. Only sequence repeats containing at least the 6 first residues of the repeat unit, i.e. CCDPCQ or similar, were counted. *, chicken EDCRP has 3 additional incomplete repeat untis. The exact number of repeat units of the tinamou is not known because of incompleteness of the gene sequence.
